# Intradermal infection and dissemination of *Candida auris* in immunocompetent and immunocompromised mouse models

**DOI:** 10.1128/spectrum.00127-24

**Published:** 2024-06-24

**Authors:** Kristine A. Towns, Abhishek Datta, Shankar Thangamani

**Affiliations:** 1 Department of Comparative Pathobiology, College of Veterinary Medicine, Purdue University, West Lafayette, Indiana, USA; 2 Purdue Institute for Immunology, Inflammation, and Infectious Diseases (PI4D), West Lafayette, Indiana, USA; Mycology Laboratory, Wadsworth Center, Albany, New York, USA

**Keywords:** *Candida auris*, skin infection, dissemination

## Abstract

*Candida auris,* an emerging fungal pathogen, predominately colonizes human skin leading to serious invasive infections in humans. Though it is assumed that skin colonization can lead to invasive infection, dissemination potential of *C. auris* from skin to internal organs is still unknown. In this study, immunocompetent and immunocompromised mouse models of intradermal skin infection were used to compare the dissemination potential of *C. auris* to internal organs. Our results suggest that *C. auris* persists in the skin tissue of both immunocompetent and immunocompromised infected mice even at 30 days post-infection. Furthermore, *C. auris* can readily disseminate from skin tissue to internal organs such as the spleen and kidney as early as 24 h post-infection and was detected until 30 days post-infection. Taken together, our findings for the first time indicate that murine skin intradermally infected with *C. auris* can readily disseminate to internal organs and cause invasive infections.

**IMPORTANCE:**

*Candida auris* is a multi-drug-resistant emerging fungal pathogen colonizes the human skin and causes life-threatening infections. However, whether *C. auris* can disseminate from the skin to internal organs is unclear. Understanding the dissemination potential of *C. auris* in both immunocompetent and immunocompromised hosts is necessary to monitor susceptible individuals and to develop novel approaches to prevent and treat this emerging fungal pathogen. Using mouse models of intradermal *C. auris* skin infection, our findings report a novel observation that mice skin intradermally infected with *C. auris* can readily disseminate to internal organs leading to systemic disease. These findings help explain the colonization, persistence, and dissemination potential of *C. auris* in immunocompetent and immunocompromised hosts and reveal that skin infection is a potential source of invasive infection.

## OBSERVATION

*Candida auris* was recently classified as an urgent threat and critical priority fungal pathogen by the US Centers for Disease Control and Prevention (CDC) and the World Health Organization (WHO) (1–3). Unlike other *Candida* species, such as *Candida albicans* which colonize the oral cavity and gastrointestinal tract, *C. auris* mainly colonizes the human skin leading to nosocomial transmission and systemic infections (4–6). Furthermore, *C. auris* enters the skin tissue and persists long term in humans and mice (4, 7). Recent findings suggest that *C. auris* was detected in murine skin tissue even after 4 months of infection and can persist for long term in the skin (4, 8). Though it was assumed that skin colonization can lead to invasive infection, to date the dissemination potential of *C. auris* from skin to internal organs is not known.

**Fig 1:**
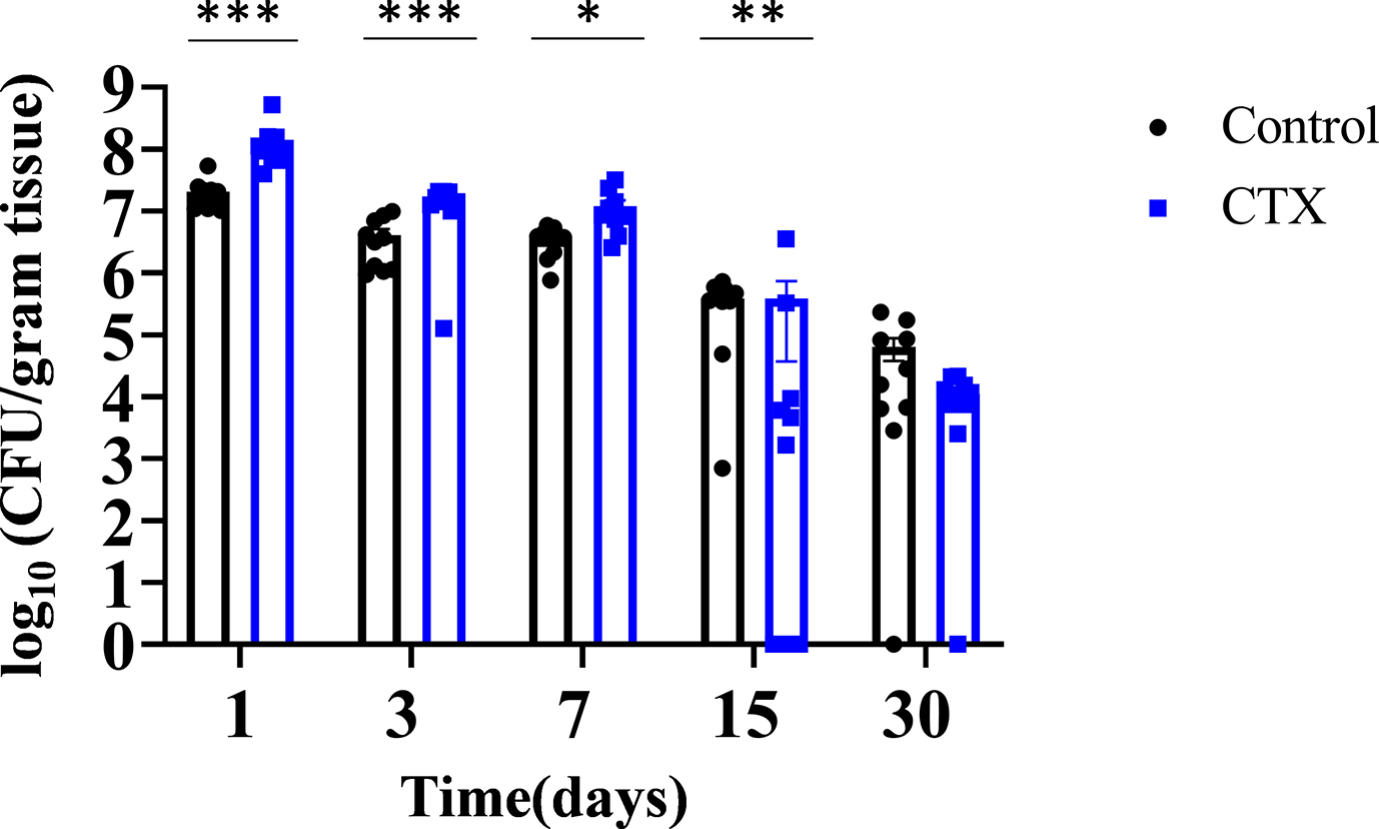
Kinetics of fungal load in mouse skin tissue treated with and without cyclophosphamide. Mice were intradermally infected with *C. auris* AR0387. The control group received only fungal inoculation and CTX group received fungal inoculation and cyclophosphamide treatment. Each group consisting of 10 mice per group were euthanized on 1, 3, 7, 15 and 30 days post-infection and skin tissue were collected. Tissues were homogenized and plated onto YPD agar containing antibiotics. Data are represented as mean ±  SEM for each group. Statistical significance was calculated using unpaired student’s *t*-test for days 1,3, and 7; Mann-Whitney U-test was used for days 15 and 30. *P ≤ 0.05, **P ≤ 0.01, and ***P ≤ 0.001 were considered as significant.

Since immunocompromised patients are highly susceptible to invasive fungal infections including *C. auris,* we utilized immunocompetent and immunocompromised C57BL/6 mouse models to compare the intradermal fungal load and the dissemination potential of *C. auris* from skin tissue to internal organs. Immunocompromised mice received cyclophosphamide 3 days prior to infection and subsequent injections once a week throughout the experiment. Untreated and cyclophosphamide-treated immunocompromised mice were intradermally infected with *C. auris* 0387 South Asian strain. Groups of untreated and immunocompromised infected mice were euthanized 1, 3, 7, 15, and 30 days post-infection to determine the fungal load in skin tissue and in internal organs such as the spleen and kidney. At early time points, 1, 3, and 7 days post-infection, mice treated with cyclophosphamide had a statistically significant higher fungal load in skin tissue (Fig. 1). Following 24 h of infection, the average fungal load in the skin tissue was 7.31 ± 0.66 log_10_ CFU/gram of tissue for untreated mice and 8.15 ± 0.76 log_10_ CFU/gram of tissue for the immunocompromised mice. After 3 days post-infection, the average fungal load in skin tissue of untreated mice was 6.61 ± 0.60 log_10_ CFU/gram of tissue, whereas cyclophosphamide-treated mice average was 7.16 ± 0.63 log_10_ CFU/gram of tissue (Fig. 1). After 7 days post-infection, the average fungal burden in the skin tissue of untreated mice was 6.55 ± 0.57 log_10_ CFU/gram of tissue in comparison with 7.08 ± 0.65 log_10_ CFU/gram of tissue in cyclophosphamide-treated mice. At 15 days, the fungal burden was 5.59 ± 0.49 log_10_ CFU/gram of tissue in untreated, infected mice compared to 5.59 ± 0.55 log_10_ CFU/gram of tissue in the cyclophosphamide-treated mice. At 30 days post-infection, the average fungal burden in the skin tissue of untreated mice was 4.80 ± 0.44 log_10_ CFU/gram of tissue, and cyclophosphamide-treated mice was 4.05 ± 0.34 log_10_ CFU/gram of tissue. We observed that cyclophosphamide-treated mice on days 1, 3, 7, and 15 post-infection did exhibit decreased healing when compared to the untreated group. These mice also exhibited visible soft tissue swelling at the infection sites that yielded purulent material on the cut surface. Taken together, *C. auris* persisted in the skin tissues of mice in both immunocompetent and immunocompromised mice until 30 days post-infection.

**Fig 2:**
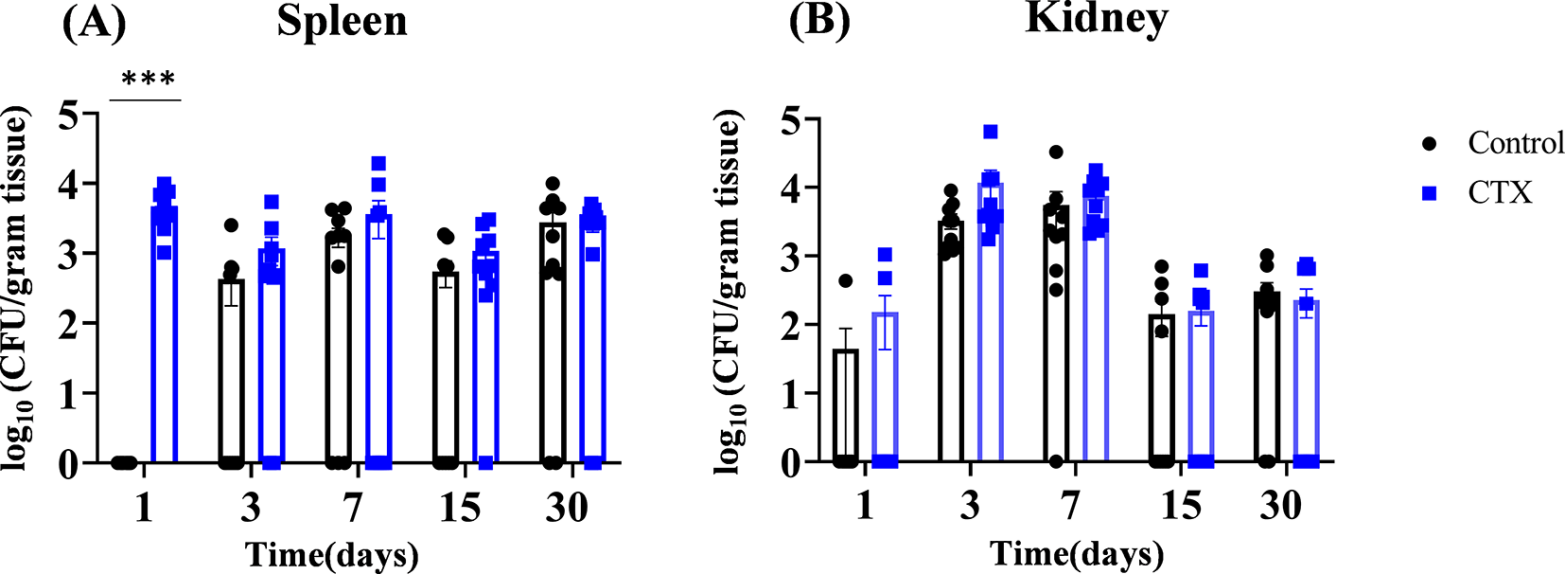
Kinetics of fungal load in internal organs from infected mice treated with and without cyclophosphamide. Groups of *C. auris* AR0387-infected mice from untreated (Control) and cyclophosphamide-treated group (CTX) were euthanized on 1, 3, 7, 15, and 30 days post-infection and internal organs such as the spleen and kidney were collected. (**A**) Spleen and (**B**) kidney were homogenized and plated onto YPD agar containing antibiotics to determine the fungal load. 10 mice per group per time point were used. Data are represented as mean ±  SEM for each group. Statistical significance was calculated using the Mann-Whitney U test, excluding day 1 for spleen data; this data set exhibited normal distribution, and significance was evaluated using an unpaired *t*-test with Welch’s approximation. ***P ≤ 0.001 was considered as significant.

Next, we examined whether *C. auris* disseminates from skin tissue to internal organs such as the spleen and kidney. Surprisingly, we identified fungal dissemination from intradermal skin to spleen and kidney in both untreated and cyclophosphamide-treated mice after 1, 3, 7, 15, and 30 days post-infection (Fig. 2). The untreated mice did not exhibit detectable levels of fungal burden on day 1 post-infection. The average fungal load in the spleen of untreated mice on 3, 7, 15, and 30 days post-infection was 2.63 ± 0.24 log_10_, 3.24 ± 0.27 log_10_, 2.74 ± 0.23 log_10_, and 3.45 ± 0.30 log_10_ CFU/gram of tissue. The average fungal load in the spleen of cyclophosphamide-treated mice was 3.67 ± 0.29 log_10_, 3.07 ± 0.27 log_10_, 3.55 ± 0.33 log_10_, 3.04 ± 0.25 log_10_, and 3.41 ± 0.27 log_10_ CFU/gram of tissue on 1, 3, 7, 15, and 30 days post-infection, respectively (Fig. 2A). Cyclophosphamide-treated mice showed 3.5 log_10_ fold higher fungal load than untreated control in spleen. All mice within the cyclophosphamide-treated group exhibited marked splenomegaly at all time points for tissue collection. At 1, 3, 7, 15, and 30 days post-infection, the average fungal load in the kidneys of untreated mice were 1.64 ± 0.16 log_10_, 3.52 ± 0.29 log_10_, 3.74 ± 0.35 log_10_, 2.15 ± 0.18 log_10_, and 2.49 ± 0.20 log_10_ CFU/gram of tissue, whereas cyclophosphamide-treated mice had 2.18 ± 0.20 log_10_, 4.07 ± 0.38 log_10_, 3.88 ± 0.32 log_10_, 2.20 ± 0.18 log_10_, and 2.36 ± 0.20 log_10_ CFU/gram of tissue. Collectively, our findings suggest that mice infected intradermally infected with *C. auris* can disseminate to internal organs such as the spleen and kidney and persist until 30 days post-infection. Given that fungal dissemination was observed in mice intradermally infected with *C. auris,* we examined if mice epicutaneously infected with *C. auris* can disseminate to internal organs. We used the epicutaneous model utilized by Huang et al. (4) and examined the fungal dissemination in both immunocompetent and immunocompromised mice. Mice were infected topically every other day three times and the fungal burdens in both the spleen and kidneys were measured on 3, 15, and 30 days post-infection. Our results identified that mice epicutaneously infected with *C. auris* did not observe detectable levels of fungal colonies in the spleen and kidney of both immunocompetent and immunocompromised groups on days 3, 15, and 30 post-infection.

Taken together, our findings suggest that *C. auris* persists in the skin tissue of untreated and cyclophosphamide-treated mice and can readily disseminate to internal organs such as the spleen and kidney. Although there is no overall trend in fungal colonization and dissemination potential of *C. auris* between untreated and cyclophosphamide-treated mice, our findings for the first time indicate that *C. auris* intradermal infection can disseminate to internal organs and potentially lead to invasive systemic infections in humans. Similar to systemic mouse models using intraperitoneal and intravenous route of *C. auris* infection that disseminate to kidneys (9, 10), our results suggest that mice intradermally infected with *C. auris* can readily disseminate to internal organs even 1 day post-infection. Moreover, the model presented by Xin et al. also suggested no statistically significant difference in overall fungal burden in the kidneys of immunocompetent and immunocompromised C57BL/6 mice 2 days post-infection (10) which agrees with our findings. Furthermore, our findings suggest that intradermal but not epicutaneous infection leads to *C. auris* dissemination to internal organs. These results suggest that tissue trauma and (or) wound infection may potentially lead to *C. auris* dissemination to internal organs.

Considering that *C. auris* preferentially colonizes human skin, this mouse model is a clinically relevant infection model to study the pathogenesis of this emerging fungal pathogen. Furthermore, this mouse model could be useful to test the effect of local and systemic antifungal therapeutics. Previous findings using the mouse epicutaneous infection model suggest that skin colonization is different among the four major clades of *C. auris* (4). Future studies using different clades and clinical isolates of *C. auris* are important to understand the dissemination potential and persistence of various clades in internal organs. We used cyclophosphamide as it is a commonly used immunosuppressive drug in cancer patients and individuals undergoing transplantation (11). However, it is critical to examine the effect of other immunosuppressive drugs in *C. auris* skin colonization, intradermal infection, and dissemination to internal organs. Collectively, our findings report a novel observation that intradermal infection of *C. auris* leads to systemic infection.

The chemicals used in this study were purchased from the following vendors: agar (BP1423-500, Fisher Bioreagents, Pittsburgh, PA USA), ampicillin (14417 Cayman Chemicals, Ann Arbor, MI USA), cyclophosphamide (CTX) (239785, Sigma Aldrich, St. Louis, MO USA), isoflurane (Dechra, Fort Worth, TX USA), phosphate buffer solution (PBS 10×) (BP3994, Fisher Bioreagents), sodium chloride (BPB58-212, Fisher Bioreagents, Pittsburgh PA USA), streptomycin (100556, MP Biomedicals, Santa Ana, CA USA), and yeast peptone dextrose (YPD) (242810, BD Difco, Franklin Lakes, NJ USA). Supplies used for this study were obtained from the following vendors: 1 mL syringes (14-826-87, BD Syringes, Fisher Scientific), 10 mL syringes (302995, BD Difco), 27 g hypodermic needles (NEZ27114, Air-Tite, VA USA), U-bottom 96-well plate (229190, Cell Treat, Pepperell, MA USA), 0.2 μm syringe filter (13-1001-06, Fisher Scientific, Pittsburgh, PA USA), veterinary trimmer (Wahl, Kent Scientific, Torrington, CT USA), chemical depilatory cream (Nair, Ewing, NJ USA), and cotton-tipped applicators (19-062-715 Puritan Medical Products, Guilford, ME USA).

C57BL/6J male and female mice from The Jackson Laboratory ranging from 6 to 8 weeks of age, housed at 28°C−29°C, 50% humidity, standard rodent diet (Teklad 2018C) and 12-h light/dark cycles. Groups of mice were infected with *C. auris* as described previously (8). One group from *C. auris*-infected mice received weekly injections of cyclophosphamide. Animal use was approved by the Institutional Animal Care and Use Committee (IACUC) at Purdue University. *Candida auris* South Asian clade strain 0387 was obtained from CDC AR Isolate Bank, USA. This strain was cultured and enriched using YPD medium at 37°C. CFU was determined using measured optical density (OD) to achieve an average of 2 × 10^7^ CFU; this suspension was used for infection at a dose of 100 µL per mouse. Mice were anesthetized with isoflurane and maintained via nosecone. Right dorsal abdominal wall was shaved and received application of depilatory cream (Nair) for 10 seconds. The skin received two additional rinses of water, blotted dry, and skin wiped clean using alcohol. A sterile 1″ 27 g needle was used to inject 100 µL of the *C. auris* isolate intradermally. Mice were placed into a clean cage in lateral recumbency for recovery.

Mice from the same strain, mixed sexes, and housing conditions as listed above were grouped into two test groups, epicutaneously infected and epicutaneously infected + CTX-treated cages. A group of 5 mice in each group was assigned a designed time point at 3, 15, and 30 days. Mice in the infected + CTX groups did receive the same CTX dose intervals as described above. *C. auris* South Asian clade strain 0387 inoculates were prepared in the same fashion as described above. The OD was measured, and a fungal load of 1 × 10^9^ CFU per mouse was then applied to the mouse skin using cotton-tipped applicators; mouse skin was prepared as described above 3 days prior to initial application to allow the skin to heal. The fungal suspension was applied every other day interval for three infection days; days post-infection were counted after the last infection day.

Cyclophosphamide 60 mg was dissolved in 3 mL of 0.9% sterile NaCl to make a 20 mg/mL solution. Once dissolved, this solution was passed through a 0.22 μm filter to produce a sterile solution, which was used no longer than 5 days per manufacturer’s instructions. The experimental group of mice received 200 mg/mL (intraperitoneal route) 3 days prior to infection with *C. auris*, and each experimental timepoint group received weekly injections, alternating left and right abdominal wall starting 7 days post-infection. Prior to weekly injections, each mouse was weighed, and doses were calculated at 150 mg/mL. Injections of cyclophosphamide were delivered using tuberculin syringes with 27 g needles. Mice within the cyclophosphamide groups were maintained in autoclaved cages, autoclaved water/food, and received weekly cage changes.

Groups of mice were euthanized via CO_2_ followed by cervical dislocation at the specified time points of 1, 3, 7, 15, and 30 days post-infection. Following euthanasia, skin tissues, spleen, and kidney were collected. Skin tissue was homogenized for 2 minutes, and internal organs were homogenized for 1 minute each in 3 mL of PBS. Homogenized suspensions were plated onto YPD agar plates with 100 μg/mL of streptomycin and 100 μg/mL of ampicillin as described before (8). Plates were cultured at 37^°^C for 24 h and the colonies were counted to determine the fungal load.

Collected data were statistically evaluated using a Mann-Whitney U-test. However, three data sets from skin samples (days 1, 3, and 7) and one data set from the spleen sample (day 1) were found to exhibit normal distribution; statistical significance was evaluated using unpaired student’s *t*-tests with Welch’s approximation. *P* values of ≤ 0.05 were considered significant. Graphical data were generated using Prism GraftPad software, and data present the median as well as standard error of the mean between immunosuppressed and immunocompetent *C. auris* groups.

## SUPPLEMENTAL MATERIAL

Supplemental figure spectrum.00127-24-s0001.docx Fig. S1.

ASM does not own the copyrights to Supplemental Material that may be linked to, or accessed through, an article. The authors have granted ASM a non-exclusive, world-wide license to publish the Supplemental Material files. Please contact the corresponding author directly for reuse.
