# Supplementary material for: Intradermal infection and dissemination of Candida auris in immunocompetent and immunocompromised mouse models
Source: Microbiol Spectr. 2024 Jun 24;12(8):e00127-24. doi: 10.1128/spectrum.00127-24 (PMC11302725; doi:10.1128/spectrum.00127-24)
Supplement: Supplemental figure — Fig. S1. [file spectrum.00127-24-s0001.docx]

Supplementary information


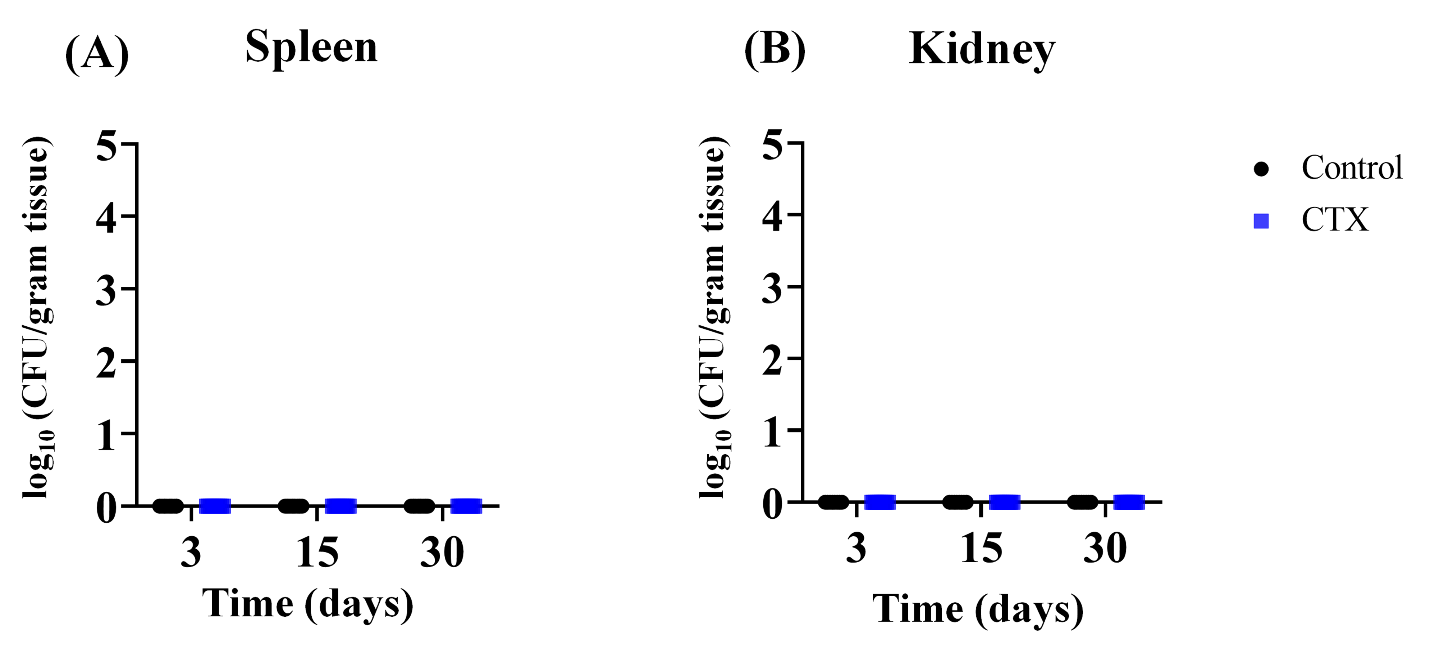


**Supplementary Figure 1.** Kinetics of fungal load in internal organs from epicutaneously-infected mice treated with and without cyclophosphamide. Groups of C. auris AR0387 infected mice from untreated (Control) and cyclophosphamide treated group (CTX) were euthanized on 3, 15- and 30-days post-infection and internal organs such as spleen and kidney were collected. **(A)** Spleen and **(B)** kidney were homogenized and plated onto YPD agar containing antibiotics to determine the fungal load. 5 mice per group per time point was used. Data are represented as mean ± SEM for each group. ​
